# AMYCO: evaluation of mutational impact on prion-like proteins aggregation propensity

**DOI:** 10.1186/s12859-019-2601-3

**Published:** 2019-01-14

**Authors:** Valentin Iglesias, Oscar Conchillo-Sole, Cristina Batlle, Salvador Ventura

**Affiliations:** 1 Institut de Biotecnologia i Biomedicina, Universitat Autònoma de Barcelona, Bellaterra, 08193 Spain; 2 Departament de Bioquímica i Biologia Molecular, Universitat Autònoma de Barcelona, Bellaterra, 08193 Spain

**Keywords:** Prion-like domain, Protein aggregation, Amyloid, Protein mutation

## Abstract

**Background:**

Around 1% of human proteins are predicted to contain a disordered and low complexity prion-like domain (PrLD). Mutations in PrLDs have been shown promote a transition towards an aggregation-prone state in several diseases.

**Results:**

Recently, we have shown that an algorithm that considers the effects of mutations on PrLDs composition, as well as on localized amyloid propensity can predict the impact of these amino acid changes on protein intracellular aggregation. In this application note, we implement this concept into the AMYCO web server, a refined algorithm that forecasts the influence of amino acid changes in prion-like proteins aggregation propensity better than state-of-the-art predictors.

**Conclusions:**

The AMYCO web server allows for a fast and automated evaluation of the effect of mutations on the aggregation properties of prion-like proteins. This might uncover novel disease-linked amino acid changes in the sequences of human prion-like proteins. Additionally, it can find application in the in silico design of synthetic prion-like proteins with tuned aggregation propensities for different purposes. AMYCO does not require previous registration and is freely available to all users at: http://bioinf.uab.cat/amyco/.

**Electronic supplementary material:**

The online version of this article (10.1186/s12859-019-2601-3) contains supplementary material, which is available to authorized users.

## Background

Prions are proteins able to adopt multiple structural conformations from which at least one has self-propagating properties [1]. Yeast prions are the best understood subset of functional prions. A common feature of most yeast prions is the presence of an intrinsically disordered and low complexity prion domain (PrD), which is necessary and sufficient for prion conversion and propagation. Proteins bearing prion-like domains (PrLDs) sharing these properties seem to exist in all kingdoms of life [2–6]. In particular, around 1% of the human proteome has been predicted to correspond to prion-like proteins [7]. This human protein subset is enriched in nucleic acid-binding proteins and involved in the formation of membraneless compartments through highly dynamic liquid-liquid demixing [7, 8]. A number of mutations in human PrLDs have been shown to convert these liquid compartments into solid aggregates, abolishing their dynamic nature and leading to the onset of neurodegenerative disorders [8, 9]. The development of tools able to anticipate the impact of such pathogenic amino acid changes is attracting increasing interest.

The self-assembling properties of prion-like proteins have been traditionally thought to rely on the biased amino acid composition of their PrLDs [10]. Disease-linked mutations would enhance the self-association of these domains, facilitating the transition to amyloid-like states [11, 12]. We have recently shown that the impact of point and multiple mutations or deletions on the aggregation of the model ALS-associated prion-like hnRNPA2 protein is best predicted by a function that takes into account both compositional features and amyloidogenic propensities [13]. Here we introduce the AMYCO (combined AMYloid and Composition based prediction of prion-like aggregation propensity) web server, which implements this approach to perform automated and fast predictions on top of prion-like protein sequences.

## Implementation

AMYCO is written in Python and uses python2.7 as the interpreter (Anaconda distribution). The web interface has been build using html/css and inputs and outputs are processed by a cgi written in perl. It all runs in a CentOS 5 server with Apache 2.2.3 using Intel Xeon ‘Clovertown’ processors.

### AMYCO pipeline

**Fig. 1:**
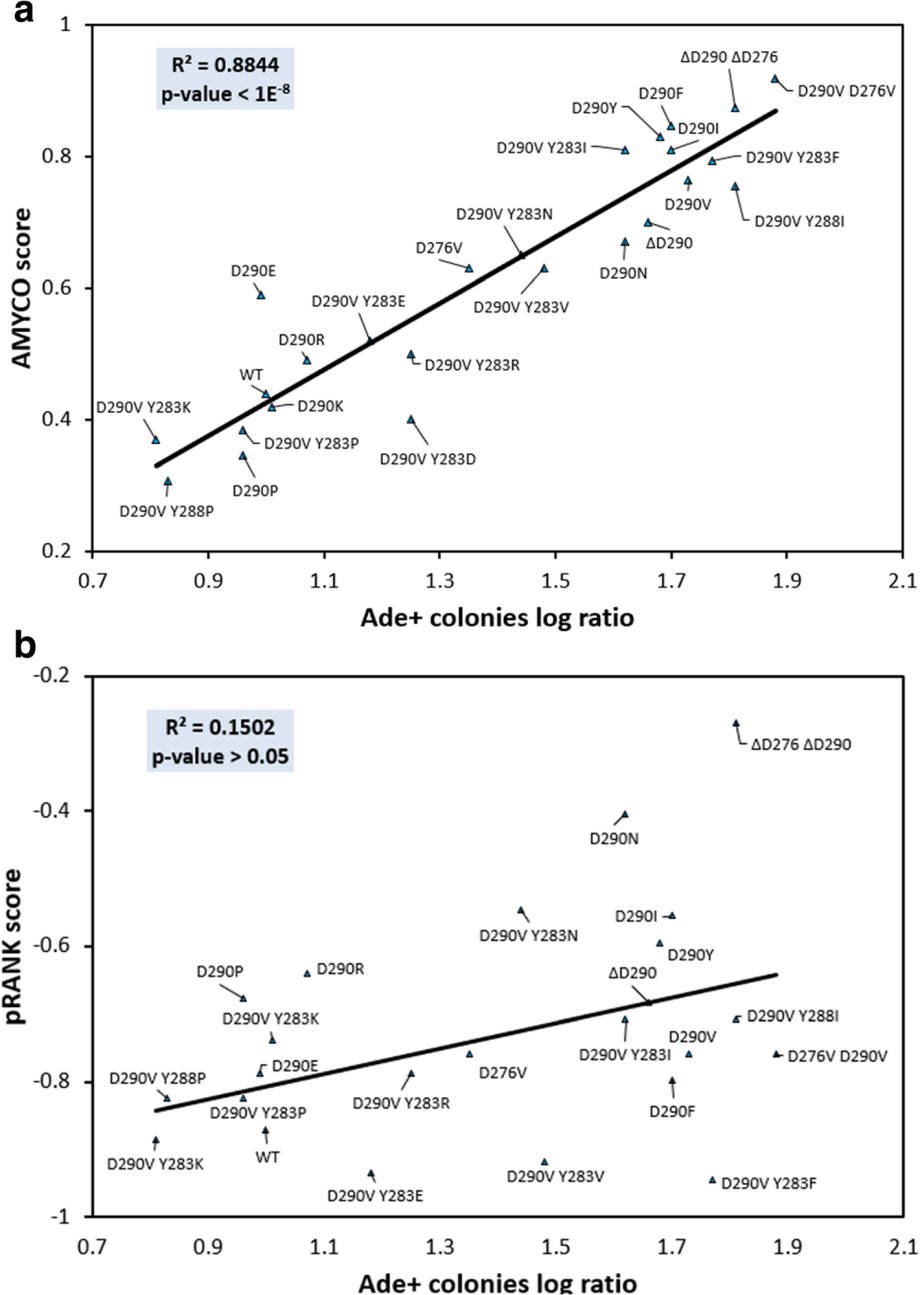
Correlation between AMYCO and pRANK predictions and the aggregation propensity of human hnRNPA2 prion-like protein variants. **a** Graphic representation of the correlation between the mutants' AMYCO (**a**) and pRANK (**b**) scores and their ability to form prionic colonies (Ade+) when expressed in yeast, a direct reporter of their aggregation propensity [13]

AMYCO evaluates the impact of mutations on the aggregation propensity of PrLDs in prion-like proteins. They can be single or multiple residues substitutions, as well as deletions and insertions. It exploits the highly significant correlation between the scores obtained from a parameterized linear function, that balances the contribution of both PrLDs composition and amyloid propensity [13], and the intracellular aggregation of hnRNPA2 variants; the unique prion-like protein for which a large set of mutations, both natural and artificial have been experimentally validated (Fig. 1a). The contribution of PrLDs amino acid composition to prion-like proteins aggregation is calculated using the PAPA algorithm [10], a program that exploits a scale of prion propensity scores for natural amino acids derived from mutagenesis experiments in the Sup35 yeast prion domain [14]. The impact of amyloidogenic sequences within the PrLDs is calculated with pWALTZ [12], a program specially intended to identify short sequences of moderate amyloid propensity able to nucleate the aggregation reaction, as those found in yeast prion domains [15]. The outputs of the two different programs are combined in a linear manner, as described in detail in the Additional file 1.

The AMYCO web server is free and open to all users, and no previous login or registration is required.

The home page of AMYCO displays three clickable links in its upper margin: (i) a help page containing a brief description of the method, the output explanation and information on examples, (ii) references for the methodology and the web application and (iii) a contact e-mail. Immediately below two links allow to switch between the *Compare Sequences* mode in which multiple sequences can be compared to a reference one, and the *Single Mutation* mode in which all possible mutations for a given protein residue are evaluated. The adjacent “Example” button fills the input text areas with the full-length sequences of wild type (*wt*) human hnRNPA2 protein and its aggregation-prone D290V mutant [16] for *Compare Sequences* mode, or all its possible mutants for position 290 for *Single Mutation* mode.

The input interface allows two working modes. In *Compare Sequences* mode (default mode); the user should introduce a reference sequence and the mutated variants (one or several) in the left and right text boxes, respectively; all in FASTA format. In the *Single Mutation* mode, the user should introduce a single sequence as well as the position to be scanned. Protein sequences should be at least 60-residues long and only the 20 standard proteinogenic amino acids are allowed.

**Fig. 2:**
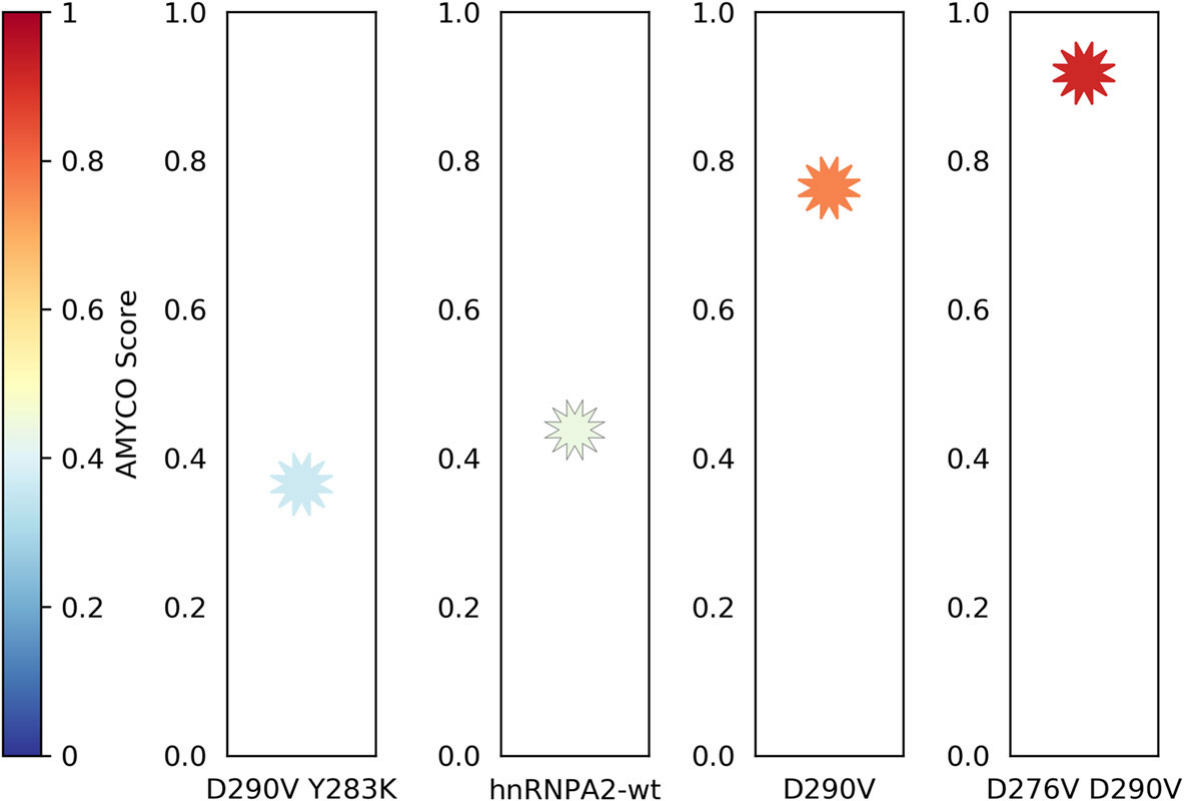
Graphical representation of the AMYCO score. AMYCO output representation of a low aggregation-prone (D290V Y283K), the *wild type*, the natural pathogenic mutant D290V and a high aggregation-prone (D290V D276V) hnRNPA2 prion-like protein variants

After submission, the output page will display a job identification number along with the names of the input sequences and the mutation position if applicable. The algorithm will return the AMYCO score for each sequence, together with a description of the mutations impact of the overall aggregation propensity. In addition, a graphical representation of the mutation/s effect will be displayed (Fig. 2). We set two arbitrary thresholds of low (< 0.45) and high (> 0.78) AMYCO scores to visualize better the overall aggregation propensities of the variants. hnRNPA2 mutants scoring < 0.45 were shown to decrease or increase < 5 times the propensity of the non-aggregating wild type protein, whereas, mutants scoring > 0.78 increased its aggregation by > 50 times [17]. Therefore, mutations rendering an AMYCO score < 0.45 are considered of low aggregation propensity and labeled in blue. Mutations that increase the aggregation propensity of the protein, but whose AMYCO score is below 0.78 are labeled in red, whereas mutations above this threshold are considered to be of high aggregation propensity are labeled in red and bold. Sequences might display AMYCO scores > 1.0, indicating that they are predicted to be more aggregation-prone than the highest scoring hnRNPA2 variant used in the parametrization of the prediction function. The output files can be downloaded for further analyses, as a ZIP file containing the resulting text explanation, a machine readable JSON file, the visualizations as a PNG file and FASTA files with the introduced sequences and the virtually generated mutants in *Compare Sequences* and *Single Mutation* modes, respectively.

## Results

### Performance

pRANK is a novel multiple-instance machine learning method aimed to predict prion propensity based on amino acid composition alone [18]. We compared the performance of AMYCO and pRANK web servers in predicting the impact of mutations on human hnRNPA2 aggregation propensity (Fig. 1). AMYCO clearly outperforms pRANK (Table 1), an observation which is consistent with the important influence that sequential features exert on protein aggregation [19].

**Table 1:** Performance of pRANK and AMYCO approaches in the prediction of mutation impact upon the aggregation of the human prion-like protein hnRNPA2

	pRANK	AMYCO
Sensitivity	0	**1**
Specificity	**1**	**1**
Precision	–	**1**
Accuracy	0.45	**1**
MCC	–	**1**
Mean % error	−7.08	**−1.25**
Standard Deviation (%)	37.71	**12.19**
SEM (%)	8.04	**2.44**
Coefficient of Determination	0.152	**0.882**
*P*-value (two tailed test)	0.468	**< 1.00E-08**
Rho (ρ)	0.334	**0.929**

The best performance according to each particular parameter is shown in bold. Details on the calculation of the different statistic parameters are provided in the Additional file 1

AMYCO was further assayed on known mutations promoting the apparition of a de novo prion-like behavior (Table 2). It was able to predict a large increase in aggregation propensity for mutations that convert the non-prionic PrLDs of PUF4, YLR177W, KC11 and PDC2 yeast proteins into prionic when expressed in yeast [20] (Table 2). Importantly, according to AMYCO, five out of the eight variants were predicted to have acquired a very high aggregation propensity. These variants are exactly the ones experimentally shown to induce a prionic phenotype with basal protein levels, without a need for overexpression [20] (Table 2).

**Table 2:** AMYCO correctly predicts prion converting mutations on yeast proteins

Protein variant	AMYCO score
PUF4 wt	0
*PUF4* ^*mut*^	*0.69*
*PUF4 ^**6PP,1N**^	**0.93**
*PUF4* ^*4PP*^	*0.60*
YLR177W wt	0
*YLR177W ^**mut**^	**0.85**
*YLR177W ^**4PP,1N**^	**1.23**
*YLR177W ^**4PP**^	**1.03**
KC11 wt	0
*KC11 ^**mut**^	**0.97**
PDC2 wt	0
*PDC2* ^*mut*^	*0.78*

AMYCO correctly predicts mutations that induce prionic phenotypes. Mutations predicted to increase and highly increase aggregation propensity are shown in italics and bold, respectively. Variants that do not need overexpression to generate a prionic phenotype in yeast are indicated with an asterisk [20]

Finally, AMYCO is able to predict an increase in aggregation propensity for a series of disease-linked mutations occurring in different human prion-like proteins. In particular, mutations in hnRNPA1 associated to ALS [16], mutations in hnRNP D0/AUF1 identified in familiar cases of Crohn Disease [21] and mutations in hnRNP DL causing limb-girdle muscular dystrophy 1G [22] (Table 3). In contrast, natural variants of these proteins bearing mutations in the PrLDs not associated to clinical phenotypes [23] do not have any significant impact in the polypeptides predicted aggregation propensity.

**Table 3:** AMYCO predicts disease-causing mutations on human prion-like proteins

Protein variant	AMYCO score
hnRNPA1 wt	0.34
*hnRNPA1 Q277K*	*0.34*
*hnRNPA1 G283R*	*0.34*
*hnRNPA1 P340S*	*0.36*
**hnRNPA1 D314V**	**0.59**
**hnRNPA1 D314N**	**0.53**
hnRNP DL wt	1.18
**hnRNP DL D378H**	**1.26**
**hnRNP DL D378N**	**1.30**
hnRNP D0 wt	1.13
*hnRNP D0 F225 L*	*1.13*
**hnRNP D0 D319V**	**1.33**
**hnRNP D0 isoform-2 D300V**	**1.33**

AMYCO identifies multisystem proteinopathy and ALS causing mutations on hnRNPA1 [16], Crohn Disease causing mutations on both isoforms of hnRNP D0/AUF1 [21] and limb-girdle muscular dystrophy 1G (LGMD1G) on hnRNP DL [22] are shown in bold. Natural variants not associated to a clinical phenotype are shown in italics

## Conclusion

AMYCO has been developed as a web application to assess the impact of mutations on the aggregation propensity of prion-like proteins, allowing a fast and accurate evaluation of the effect of disease-associated mutations in these polypeptides; as well as engineering novel variants with designed aggregation propensities for different applications.

## Availability and requirements

Project name: AMYCO.

Project home page: http://bioinf.uab.cat/amyco/.

Operating system(s): Platform independent.

Programming language: A computing core coded in Python and a front end written in a combination of html and perl cgi.

Other requirements: A web browser with a working internet connection.

License: None.

Any restrictions to use by non-academics: None.

## Additional file

Additional file 1: Dataset obtention and performance analysis. (PDF 219 kb)

## Abbreviations

MCC: Matthews correlation coefficient
PrD: Prion domain
PrLD: Prion like domain
SEM: Standard error of the mean
