# Supplementary material for: AMYCO: evaluation of mutational impact on prion-like proteins aggregation propensity
Source: BMC Bioinformatics. 2019 Jan 14;20:24. doi: 10.1186/s12859-019-2601-3 (PMC6332698; doi:10.1186/s12859-019-2601-3)
Supplement: Supplementary file 1 — Dataset obtention and performance analysis. (PDF 219 kb) [file 12859_2019_2601_MOESM1_ESM.pdf]

Additional file to:

AMYCO: Evaluation of mutational impact on prion-like proteins aggregation propensity

Dataset and scoring function

Ross and co-workers [1] analysed the aggregation propensity of wild type (wt) hnRNPA2 and a set of 24 different PrLD mutants by replacing the yeast Sup35 PrD by the PrLD of the different hnRNPA2 variants and assessing their ability to maintain the prion [PSI+] phenotype in yeast. The aggregation propensity was assessed by evaluating the number of cells displaying a prionic behaviour for each hnRNPA2. In a recent work we exploited these data to quantify the extent to which each mutant increases/decreases the aggregation propensity of the hnRNPA2 PrLD relative to the wt sequence [2]. According to this normalization, the aggregation propensity in the 24 mutant set ranged from 0.3-fold to 245-fold the one exhibited by the wt protein. Only mutations increasing the proportion of prionic cells, relative to wt, by more than one order of magnitude ($\log_{10} > 1$) where considered positive (13 sequences) and the rest were taken as negative (11 sequences) for the calculations in Table 1.

AMYCO corresponds to a linear combination of normalized PAPA [3] and pWALTZ [4] scores. Individual PAPA and pWALTZ scores for the 25 proteins set were normalized between 0 and 1 (PAPAn and pWALTZn). AMYCO score = 0.5*PAPAn + 0.5*pWALTZn. The AMYCO scores were normalized between 0 and 1 for the complete mutation dataset (AMYCOn) and the positive detection threshold set at 0.63, which exhibited the best discrimination power according to a ROC curve.

In its original implementation, PAPA defines the boundary of its prion-like domains as 41 consecutive 41 amino acid windows; which translates into an 81-residue window for any analysed positive sequence [3]. This length is shorter than that of the PrDs of many *bona fide* yeast prions, which according to their annotation in Uniprot [5], range between 85 and 325 residues. We rationalized that multiple 81 PAPA prion-domain windows could overlap and generate longer PrLDs of diverse lengths. Accordingly, AMYCO considers a PrLD the sequence comprising all consecutive 41-residue windows scoring above the PAPA threshold, without defining a strict limit in length. Is this PrLD sequence that is afterward used to identify and score short amyloidogenic stretches with pWALTZ [4]. Because in the original pWALTZ implementation the contribution of Pro residues were arbitrarily excluded from calculations, AMYCO evaluates the impact of mutations to Pro using only the PAPAn score.

Performance analysis

The sensitivity, specificity, precision, accuracy and Matthews correlation coefficient (MCC) were calculated from point mutations in hnRNPA2 considering positive those mutations which increased the mutant/*wild type* prion colony ratio by at least 10-fold. The dataset was composed of 13 True positives (TN) and 11 true negatives (TN). For evaluation purposes we abbreviate false positives as FP and false negatives as FN. Statistical measures were calculated as follows: Sensitivity=TP/(TP+ FN)

Specificity=TN/(TN+FP)

Precision=TP/(TP+FP)

Accuracy=(TP+TN)/(TP+TN+FP+FN)

$MCC=(TP*TN-FP*FN)/((TP+FP)(TP+FN)(TN+FP)(TN+FN))^{1/2}$.

Error calculations were performed by subtracting experimental results from predictions. Experimental error percentage was calculated for each sample and each predictor. The mean % error for each predictor was obtained by dividing it into the number of samples. The standard deviation and standard error of the mean (SEM; in percentage) were also obtained.

Finally, Pearson's coefficient of determination, Spearman's Rho (ρ) and two-tailed p-value were calculated from the regression analysis for each predictor against the prionic colonies log ratio [6, 7] (**Figure 1**).

References:

1. Paul KR, Molliex A, Cascarina S, Boncella AE, Taylor JP, Ross ED: **Effects of Mutations on the Aggregation Propensity of the Human Prion-Like Protein hnRNPA2B1**. *Mol Cell Biol* 2017, **37**(8).
2. Batlle C, Fernandez MR, Iglesias V, Ventura S: **Perfecting prediction of mutational impact on the aggregation propensity of the ALS-associated hnRNPA2 prion-like protein**. *FEBS letters* 2017.
3. Toombs JA, Petri M, Paul KR, Kan GY, Ben-Hur A, Ross ED: **De novo design of synthetic prion domains**. *Proc Natl Acad Sci U S A* 2012, **109**(17):6519-6524.
4. Sabate R, Rousseau F, Schymkowitz J, Ventura S: **What makes a protein sequence a prion?** *PLoS Comput Biol* 2015, **11**(1):e1004013.
5. UniProt C: **UniProt: a hub for protein information**. *Nucleic Acids Res* 2015, **43**(Database issue):D204-212.
6. **p-Value Calculator for Correlation Coefficients** [http://www.danielsoper.com/statcalc]
7. **Social Science Statistics** [https://www.socscistatistics.com/]
